# The National Baby Week Council

**Published:** 1920-10-16

**Authors:** 


					The National Baby Week Council*
Comic if wT .Cn,T' Meeti?ff of the National Baby
Street \Y 1 1? n ,at Arimtage "all, 224 Great P?rtl"!
Blind)' ,,, t ! V by tho National Institute for
Sybil VI ' r V' October 26, 1920, at 3 p.m. : Chain')""'
Mr II ()?'"L<>SS '^',on('^a- An address will bo given
Iloaltli ' I.-., ,U!f bury' Assistant Secretary, Ministry0
What Bah w Th? Futuro ?f Infant Welfare Work
to l?o foil YlC Ca" db to Ilromoto It." The address '
kindly promispd^ a ''lscuss'?n in which the following' jj
4? part: Sir James Cantlie, KA-^'
A resolnKr/ '? Sloan Chesser, and Mrs. H. 13. Ir ,? n
will bo prcsoiil)r?PiOSCTfbv ^10 Viliago Councils FodL-ra ^j
I ron.in/^'hy ?fiM Churton (Secretary to thecal
Baby Wool- r< ' *i That this meeting of tho Na, ^ e
Minfstor of b T' d?sircs to caI1 the attention ?f H?.
,nges for f)-o r ? t|?? 8?noraI liack of provision J cofl.
stitutes a lh-bvaT5, refusp and night-soil, whlC, urgos
that prewiir\> .u i^r ,to tho ho,llth of children,
to deal with Sfl' i bought to bear on local auti f j
may bo K e matter m their district." Tickets (
- ix> had on application to the Secretary, N.B.W 0-

				

